# Inter-rater reliability of the Abbreviated Injury Scale scores in patients with severe head injury shows good inter-rater agreement but variability between countries. An inter-country comparison study

**DOI:** 10.1007/s00068-022-02059-x

**Published:** 2022-08-16

**Authors:** Amy C. Gunning, Menco J. S. Niemeyer, Mark van Heijl, Karlijn J. P. van Wessem, Ronald V. Maier, Zsolt J. Balogh, Luke P. H. Leenen

**Affiliations:** 1 Department of Trauma Surgery, University Medical Center Utrecht, Suite: G04.228, Heidelberglaan 100, 3584 CX Utrecht, The Netherlands; 2 Department of Trauma Surgery, Harborview Medical Center, University of Washington, Seattle, WA USA; 3 Department of Traumatology and Surgery, John Hunter Hospital and University of Newcastle, Newcastle, NSW Australia

**Keywords:** Inter-rater reliability, Abbreviated Injury Scale, Inter-country comparison, Level I trauma centers, Scoring variability

## Abstract

**Introduction:**

Substantial difference in mortality following severe traumatic brain injury (TBI) across international trauma centers has previously been demonstrated. This could be partly attributed to variability in the severity coding of the injuries. This study evaluated the inter-rater and intra-rater reliability of Abbreviated Injury Scale (AIS) scores of patients with severe TBI across three international level I trauma centers.

**Methods:**

A total 150 patients (50 per center) were randomly selected from each respective trauma registry: University Medical Center Utrecht (UMCU), the Netherlands; John Hunter Hospital (JHH), Australia; and Harborview Medical Center (HMC), the United States. Reliability between coders and trauma centers was measured with the intraclass correlation coefficient (ICC).

**Results:**

The reliability between the coders and the original trauma registry scores was 0.50, 0.50, and 0.41 in, respectively, UMCU, JHH, and HMC. The AIS coders at UMCU scored the most AIS codes of ≥ 4. Reliability within the trauma centers was substantial in UMCU (ICC = 0.62) and HMC (ICC = 0.78) and almost perfect in JHH (ICC = 0.85). Reliability between trauma centers was 0.70 between UMCU and JHH, 0.70 between JHH and HMC, and 0.59 between UMCU and HMC.

**Conclusion:**

The results of this study demonstrated a substantial and almost perfect reliability of the AIS coders within the same trauma center, but variability across trauma centers. This indicates a need to improve inter-rater reliability in AIS coders and quality assessments of trauma registry data, specifically for patients with head injuries. Future research should study the effect of differences in AIS scoring on outcome predictions.

## Introduction

One of the primary goals of the American College of Surgeons Committee on Trauma (ACS-COT) is to have trauma registries in trauma centers that contain detailed, reliable, and readily accessible data [1]. These data make national and international comparative assessments of trauma patients possible. For this, uniform datasets and data definitions are of major importance.

In our previous study, a substantial difference in mortality of patients with severe traumatic brain injury (TBI) was demonstrated across three international level I trauma centers [2]. The differences in this study could not only be attributed to different treatment strategies, but also to the variability in the severity of the injury scores.

According to the ACS-COT, all injuries are scored according to the Abbreviated Injury Scale (AIS) score. The AIS score was introduced and implemented in global trauma care in the 70 s [3]. It is an anatomically based, consensus derived, global severity scoring system that classifies each injury by body region according to its relative importance. The score ranges from 1 to 6 on an ordinal scale; the higher the score, the more severe the injury. The highest scores from the three most severely injured body regions are squared and summed to obtain the Injury Severity Score (ISS) [3, 4]. Both the AIS and the ISS scores have been extensively used in several trauma scores and trauma care analysis [5–9]. A rater variability in these scores across trauma centers might cause differences in outcome analyses.

Only a few studies have evaluated the variability of the AIS scores; between educated coders within a hospital, different AIS coding versions or between different specialties in a hospital [10–17]. None of the studies addressed the rater variability in the AIS coding across trauma centers in different international trauma systems.

Therefore, the aim of the present study is to evaluate the inter- and intra-center rater reliability of AIS scores of patients with severe TBI across three level I trauma centers in different countries.

## Methods

### Study design

This multi-center study was performed in three level I trauma centers from different countries: the University Medical Center Utrecht (UMCU), Utrecht, the Netherlands, John Hunter Hospital (JHH), Newcastle, Australia, and Harborview Medical Center (HMC), Seattle, Washington, USA.

The study was performed following the Guidelines for Reporting Reliability and Agreement Studies (GRRAS) [18].

This study was approved, and waivers of consent were provided by the Institutional Review Board of the UMCU, JHH, and HMC (reference number: WAG/mb/12-404). The study was conducted in accordance with the principles of the Declaration of Helsinki and Good Clinical Practice Guidelines [19, 20].

### Patient selection

Patients were selected from an extensive database of which a detailed description and key demographic and trauma center differences were described in our previous study [2].Our previous study demonstrated a difference in mortality in the patients with a head AIS score of 4 and higher [2].

Patients were randomly selected out of the three institutional trauma registries from the year 2012 to minimize recall bias. Selections included directly admitted trauma patients, aged 18 years and older with an AIS score of 3 or higher in the head region. Cases excluded from selection were patients dead on arrival in Emergency Department (ED) (i.e., no vital signs at ED presentation or those who died within 30 min), major burns (> 20% of total body surface area), and if transferred to another hospital.

A total of 150 patients were randomly selected by the computer without replacement. This sample size rendered a statistical power of 80%, with a confidence interval of 95%, and a population proportion of 61% (the mean population proportion of all three centers). For both UMCU and JHH, this sample size has a margin of error of 5%, and the margin of error for HMC was 7%. We decided to accept this small difference and not to increase the sample size from the HMC database in order to keep the selected number across the centers equal and minimize recall and selection bias.

### AIS coders

Both UMCU and HMC have three trained AIS coders. In UMCU, all coders completed an AIS course provided by the Dutch National Trauma Registry and attend national user group meetings four times a year. The AIS coders in HMC have all completed the AIS coder training from the Association for the Advancement of Automotive Medicine. JHH has five trained AIS coders. The coders are trained through a central teaching system in New South Wales. Coders had AIS coding experience varying between 2 months and 20 years with a median of 6 years.

Each coder was requested to code head injuries of the randomly selected sample. They all used the AIS score version 2005. Coding started in 2016 and was performed independently from each other. The coders were blinded for the registered AIS score and patient origin in the provided datasets.

### Data collection

All relevant radiology reports, admission reports, discharge reports, prehospital information, and other relevant patient notes addressing the TBI were gathered. The Dutch reports were translated in English by for the AIS coders in JHH and HMC by AG and LL, skilled in both medical Dutch and English. The Dutch AIS coders used the English reports from JHH and HMC.

### Outcome variables and statistical analysis

The primary outcome measure was the agreement among coders on AIS severity. To quantify agreement between coders and centers, the weighted kappa for ordinal variables was measured with a two-way mixed, single-measures, intraclass correlation coefficient (ICC). This model was chosen, following literature guidelines, because we have a specific sample of raters for all subjects and we have selected a single measurement, the highest AIS score from each center, for the measurement of the ICC between the trauma centers [21, 22]. Secondary outcome measures were the inter- and intra-center agreement among AIS coders. Additionally, possible overestimation of patients with severe injury was measured. Therefore, we explicitly also included patients with an AIS score of 3 (moderate injury). The ICC was calculated between AIS coders from the same trauma center and across the trauma centers. The arbitrary classification of Landis and Koch was used to classify the agreement (Table 1) [23].

**Table 1:** Guidelines for agreement measures

0.00–0.20	Slight
0.20–0.40	Fair
0.40–0.60	Moderate
0.61–0.80	Substantial
0.81–1.00	Almost perfect

Landis JR, Koch CG. Biometrics 2008

For each patient the highest AIS severity in the head region was selected and presented in frequencies. The highest AIS severity given to the patient by coders of the same hospital was selected to compare differences between the centers. Averages of total frequencies were used to correct for the unequal number of coders per center.

The analyses were performed for the overall dataset and separated for each country-specific dataset. The statistical analyses were performed with SPSS, version 20.0 (IBM Corp., Armonk, NY) for Windows. Significance of statistical differences was attributed to *p* < 0.05.

## Results

An overview of the frequencies of the highest severity score in the head region of the selected patients is shown in Table 2. The AIS coders at UMCU scored the most AIS codes of ≥ 4, with an average frequency of 92 between the three AIS coders, followed by the coders from HMC (average = 70) and JHH (average = 70) (Table 3). Altogether, the three AIS coders from UMCU scored the injuries of 39 patients with a severity below 3. A total of 46 patients were scored with a severity below 3 by the three AIS coders in JHH, and 29 patients by the two AIS coders in HMC (Table 3). Only one AIS coder scored all patients, while the other coders scored some patients with a ‘zero’ (no head injury) or ‘not applicable’ because they felt that there was no trauma preceded or the trauma was too long ago.

**Table 2:** Frequencies of the severity of the original AIS scores in the trauma registry

	3	4	5	6
UMCU	15	23	12	–
JHH	16	24	9	1
HMC	15	21	14	–

**Table 3:** Frequencies of AIS score severity

	1	2	3	4	5	6
UMCU						
Coder 1	5	11	47	48	34	1
Coder 2	–	8	32	63	41	–
Coder 3	3	12	45	59	31	–
JHH						
Coder 1	2	13	60	32	38	1
Coder 2	-	16	64	30	37	–
Coder 3	1	14	60	32	40	–
HMC						
Coder 1	1	16	56	35	37	–
Coder 2	3	9	68	28	38	1

The overall reliability between the AIS coders in UMCU was substantial (ICC = 0.62), ranging from ICC = 0.55 in the HMC dataset to ICC of 0.67 in the UMCU dataset. The AIS coders from JHH had an almost perfect reliability (ICC = 0.85), ranging from an ICC of 0.78 in the HMC dataset and ICC = 0.90 in both UMCU and JHH dataset. In HMC, the overall reliability was substantial (ICC = 0.78), ranging from 0.71 in the HMC dataset to 0.83 in the JHH dataset (Table 4).

**Table 4:** Intraclass correlation of AIS coders within same trauma centers

	UMCU	JHH	HMC
Overall dataset	0.62	0.85	0.78
UMCU dataset	0.67	0.90	0.77
JHH dataset	0.63	0.90	0.83
HMC dataset	0.55	0.78	0.71

The overall reliability between the trauma centers was the highest between the AIS coders from UMCU and JHH, and JHH and HMC, with both an ICC = 0.70. Separated in country-specific datasets the reliability was almost perfect between JHH and HMC in the UMCU dataset (ICC = 0.84). The overall reliability was lowest between the AIS coders from UMCU and HMC, with the lowest ICC in the HMC dataset (ICC = 0.52) (Table 5).

**Table 5:** Intraclass correlation of highest overall AIS code between centers

	UMCU vs JHH	UMCU vs HMC	JHH vs HMC
Overall dataset	0.70	0.59	0.70
UMCU dataset	0.58	0.65	0.84
JHH dataset	0.78	0.55	0.56
HMC dataset	0.63	0.52	0.77

In Table 6, an overview of the correlations between two individual AIS coders within and across the trauma centers is presented. No clear outliers exist between the AIS coders.

**Table 6:** Intraclass correlation between all AIS coders

	UMCU			JHH			HMC	
	Coder 1	Coder 2	Coder 3	Coder 1	Coder 2	Coder 3	Coder 1	Coder 2
UMCU								
Coder 1	–	–	–	–	–	–	–	–
Coder 2	0.59	–	–	–	–	–	–	–
Coder 3	0.66	0.62	–	–	–	–	–	–
JHH								
Coder 1	0.68	0.54	0.74	–	–	–	–	–
Coder 2	0.64	0.54	0.69	0.83	–	–	–	–
Coder 3	0.65	0.58	0.76	0.88	0.85	–	–	–
HMC								
Coder 1	0.69	0.60	0.65	0.64	0.68	0.65	–	–
Coder 2	0.68	0.59	0.60	0.69	0.68	0.66	0.78	–

## Discussion

To our knowledge, this is the first study measuring inter- and intra-rater reliability of AIS scores on an international level. The overall reliability within the trauma centers was substantial in UMCU (ICC = 0.62) and HMC (ICC = 0.78), and almost perfect in JHH (ICC = 0.85). We observed a variability in the highest overall AIS scores between trauma centers, with ICCs ranging from 0.52 to 0.84.

In this study, the distribution of the original AIS score severity (stratified in < 4 and ≥ 4) was similar. Nevertheless, the UMCU coders scored more patients with a severity of 4 and higher compared to JHH and HMC. There is no clear explanation for the variability between and within trauma centers. All AIS coders were trained with a course recognized on at least a national scale. The participants employed the 2005 edition of the AIS coding handbook as it was the only version that all the participants were certified for and trained in at the time. Most of the participating centers started using the 2008 version since 2015, and thus the updated AIS codes were not available for the original dataset. While the disparities are limited between the two versions, it is not yet known if this would significantly impact severity assessment and subsequent ISS calculations [24]. There still might be a difference in the process of training and maintaining expertise between the centers that could influence the reliability. Furthermore, the experience of the AIS coders varies between the trauma centers. Years of experience could play a role in the agreement between AIS coders; however, the data of this study do not demonstrate this (data are not shown for anonymity of the participants). Nonetheless, the reliability of the individual AIS coder could be measured in intra-rater reliability. It would be interesting to investigate the impact of a difference in AIS scores on the outcome predictions in future studies.

The reassessment of our sample differed significantly from the hospitals’ registries. Our sample only included cases with an AIS severity head region ≥ 3 of which a substantial number of patients were re-coded with a lower severity score. This demonstrates that numerous injuries might have been overestimated in the original trauma registry or underestimated in our sample. There is no gold standard available for AIS coding; therefore, some influence of subjectivity will always be present. However, overestimation of injury severity is not without consequence. Significant variability in AIS severity scoring might have a substantial influence on the calculated probability of survival, as the Injury Severity Score (ISS) is one of its depending parameters [5]. A study by Maduz et al. also showed significant variability on reassessment of AIS severity in 16% of their sample, resulting in a 24% these patients who were misclassified as polytrauma patients (ISS ≥ 16) [25]. Systematic variability could result in inaccurate outcome predictions between trauma populations. Consequently, this could lead to the misassumption that trauma centers are performing worse than another.

Several studies address the reliability of the AIS scores in terms of ICC or (weighted) kappa statistics in comparative literature. These studies have compared scores between a computer system and human coders, and evaluated the intra-rater differences after a training course, and between AIS coders working with the same trauma registry [10, 12, 15–17]. The scores in these studies evaluating the agreement between the AIS scores vary from a kappa score of 0.79 to an agreement of the AIS codes of 39% [10, 16]. These data are comparable to our findings.

Our study also had several limitations. Firstly, despite the extensive training programs, scoring injuries with AIS remain a subjective evaluation. The introduction of a standardized quality assessment process for trauma registries will help maintain high quality data in the registries. Currently, there is no uniform design how to perform these quality assessments. Olthof et al. developed a format for these studies which showed to be feasible and could be used as a basis to develop such a methodology [17].

Secondly, reassessment of the AIS scores is time and resource demanding. Although meeting the pre-calculated sample size, the sample was limited by feasibility for the participants. This possibly could have led to a degree of variability, where a larger sample size would have reduced the overall variation. However, a general rule of thumb in biostatistics is “do more less well” where spending time and resources to improve the precision of individual measurements is unreasonable in practically all research where the emphasis is on biological outcomes [26]. So rather than refining the AIS of the head as an instrument for comparison of limited samples, it may be best reserved for use in more substantial trauma cohorts.

Lastly, the English and thus foreign language medical records could influence the greater variability within the UMCU AIS coders in comparison with the JHH and HMC coders. UMCU coders used the original English records for the severity coding of the JHH and HMC dataset. In contrast to the JHH and HMC coders who received the UMCU medical records translated to English. Although English being the secondary language in the Netherlands, it could have influenced our results slightly. Finally, some recall bias might have occurred during the scoring, as AIS coders could have recognized cases and AIS codes. However, a significant recall bias would be unlikely as the timing between the original scoring and the scoring of this study was at least 3.5 years.

### Implications

The AIS scores belong to the most important parameters in trauma care which are used to evaluate injury patterns and characteristics in trauma populations; they are extensively used in comparison studies of injury outcomes across hospitals. Future research should not only evaluate the reliability and reproducibility of AIS codes of the head region but also focus on improving these measurements by, e.g., standardized (international) education. Furthermore, it may well be that brain injured patients have decisive and discerning subpopulations for which the AIS in its current use as an instrument may be ill-suited.

## Conclusion

The results of this study demonstrated a substantial and almost perfect reliability of the AIS coders within the same trauma center. However, across trauma centers the reliability was variable between the AIS coders and one should be aware of overestimation. These results indicate that there is much room for improvement in the reliability between AIS coders. In future studies, the impact of differences in AIS scoring on the outcome predictions should be investigated. Furthermore, quality assessments of trauma registry data should be implemented and performed routinely as they provide the essential information for clinicians and policymakers alike.

## Data Availability

The dataset supporting the conclusions of this article are available upon reasonable request from the corresponding author.
